# Rapid increase of MFGE8 secretion from endometrial epithelial cells is an indicator of extracellular vesicle mediated embryo maternal dialogue

**DOI:** 10.1038/s41598-024-75893-1

**Published:** 2024-10-29

**Authors:** Subhashini Muhandiram, Suranga Kodithuwakku, Kasun Godakumara, Alireza Fazeli

**Affiliations:** 1 Institute of Veterinary Medicine and Animal Sciences, Estonian University of Life Sciences, Kreutzwaldi 62, Tartu, 51006 Estonia; 2 Department of Animal Science, Faculty of Agriculture, University of Peradeniya, Peradeniya, 20400 Sri Lanka; 3 Department of Pathophysiology, Institute of Biomedicine and Translational Medicine, University of Tartu, Ravila St. 14B, Tartu, 50411 Estonia; 4 Division of Clinical Medicine, School of Medicine & Population Health, University of Sheffield, Sheffield, S10 2RX UK

**Keywords:** Embryo, Endometrium, Extracellular Vesicles, Milk fat globule protein 8, Embryo Implantation, Cell biology, Nanoparticles

## Abstract

Successful embryo implantation relies on synchronized dialog between the embryo and endometrium, and the role of extracellular vesicles (EVs) in facilitating this cross-talk has been recently established. In our previous study, milk fat globule-EGF factor 8 protein (MFGE8) was identified as increasing in receptive endometrial epithelial cells (EECs) in response to trophoblastic EVs. However, the dynamics of MFGE8 protein in this context are not completely understood. Therefore, we examined its expression and secretion in EECs exposed to estrogen, progesterone, and trophoblastic EVs to gain deeper insights into its potential as an indicator of EV-mediated embryo-maternal dialogue. Our findings revealed that MFGE8 secretion is sensitive to estrogen and progesterone, and that trophoblastic EVs stimulate their release in both receptive and non-receptive EECs. Furthermore, trophoblast EV function was dose and time-dependent. Notably, the secretion of MFGE8 increased within a short timeframe of 30 min after addition of EVs, suggesting the possibility of rapid processes such as binding, fusion or internalization of trophoblastic EVs within EECs. Interestingly, MFGE8 released from EECs was associated with EVs, suggesting increased EV secretion from EECs in response to embryonic signals. In conclusion, increased MFGE8 secretion in this embryo implantation model can serve as an indicator of EV-mediated embryo-maternal dialogue.

**Supplementary Information:**

The online version contains supplementary material available at10.1038/s41598-024-75893-1.

## Introduction

Embryo implantation is crucial for establishing pregnancy in mammals. In humans, it is a highly co-ordinated process, where the embryo first approaches the embryo homing site, adheres, and eventually invades the uterine luminal epithelium^1^. Around 75% of the early pregnancy losses in humans can be attributed to embryo implantation failure^2^. Despite the rapid progress in development of assisted reproduction technologies (ARTs), such as in vitro fertilization and embryo transfer, their average success rate remains modest, hovering around ~ 30%^3^. While the quality of the embryo notably affects the outcomes of ARTs, synchronized crosstalk between embryo and maternal environment is also indispensable for orchestrating the steps necessary for a successful pregnancy^4^. Despite extensive research, the precise molecular events underpinning the embryo maternal dialogue remain elusive, posing challenges to the advancement of ARTs and infertility treatments^5^.

Interaction between the blastocyst and endometrium can only occur during a short timeframe of the human menstrual cycle, namely the “window of implantation”. During this critical period, the endometrium transforms into an optimally receptive state, preparing to support embryo attachment. Two main ovarian hormones, estrogen and progesterone^6,7^, are known to regulate the endometrial receptivity for embryo implantation along with other bioactive components. The initial contact between the embryo and the receptive endometrium triggers a dynamic and coordinated exchange of signals at the feto-maternal interface. Part of this molecular signalling is governed by growth factors and various other cytokines^8–13^. Recently, EVs emerged as one of the key communication channels that transfers signals between the embryo and endometrium. EVs are a group of nano-sized vesicles that play a vital role in intercellular communication by transporting bioactive substances such as, proteins, nucleic acids, and lipids to recipient cells. They are involved in various physiological and pathological processes^14,15^. EVs carry a unique molecular fingerprint that reflects the characteristics and molecular state of their parent cells^16^. Although the biological effects of the EVs are thought to result from the functional delivery of specific molecular cargo, the exact mechanisms by which recipient cells read or interpret EV messages are not fully understood.

Multiple studies have highlighted the role of embryonic EVs in the embryo-maternal crosstalk during embryo implantation^17–20^. In a previous study, we reported that trophoblast-derived EVs can modulate significant transcriptomic changes in endometrial epithelial cells (EECs) in vitro^19^. Remarkably, these changes initiated as early as 30 min of EV treatment, indicating the existence of a rapid mechanism of signal transfer from embryo to EEC via EVs^21^. In corroboration with the above findings, we recently observed that EVs from trophoblast cells can also stimulate EECs to secrete protein molecules necessary for embryo implantation^22^. Moreover, mounting evidence supports the bidirectional nature of EVs based communication in mediating embryo attachment. For example, endometrial-derived EVs have been demonstrated to support embryo attachment by enhancing adhesive, invasive, and developmental capacities of blastocysts^18,23,24^. Such interactions between the embryo and endometrium mediated via EVs can occur through diverse mechanisms. The steps of EV mediated cellular interactions typically include targeting the cell, binding of the EV to the cell^25^, release of the cargo content through cellular uptake^26,27^ or EV fusion^28,29^. Although not all steps are necessarily required, one common notion regarding the EV mode of action is the EV cargo content release upon EV uptake and initiation of signal transduction. Interestingly, embryonic and endometrial EVs have been shown to contain unique cargo components, indicating their potential to transmit favourable signals that facilitate successful embryo attachment to the endometrium through delivery of their cargo. For instance, blastocysts are known to secrete EVs containing a variety of miRNA species^30^. These miRNAs target EECs and stromal cells to potentially mediate functions, such as cell adhesion and migration^31^. Similar to miRNAs, both embryonic and endometrial EVs are enriched in proteins that have implications in the process of embryo implantation^32^. Nevertheless, the exact molecules and mechanisms involved in trophoblast-derived EV signalling and the dynamics of the trophoblast-derived EVs effect on EEC protein changes are yet to be elucidated.

We recently identified MFGE8 (Human Lactadherin) as a protein that increases in the receptive EEC secretome in response to trophoblast-derived EVs^22^, and interestingly, it appears to play a significant role in the context of embryo implantation^33–35^. MFGE8 is a cysteine-rich secretory glycoprotein that shows bivalent binding activity towards acidic phospholipids and integrin receptors^36^. It displays cyclic changes and altered expression from the proliferative to mid-secretory phase of the menstrual cycle in humans. Therefore, intracellular MFGE8 protein expression in EECs appears to be associated with endometrial receptivity^37–39^. Estrogen and progesterone are the two main steroid hormones responsible for setting up the endometrial receptivity. However, the roles of estrogen and progesterone in MFGE8 protein expression and secretion have not been clearly demonstrated. Therefore, in the current study, we investigated the effects of estrogen and progesterone hormone combinations that mimic the luteal and proliferative phases of the menstrual cycle on MFGE8 protein expression and secretion from EECs.

To better understand the potential mechanisms associated with MFGE8 secretion in EECs, we studied MFGE8 protein secretion dynamics in EECs in response to trophoblast-derived EVs, as a function of time and concentration. Increased MFGE8 secretion wasswift and dose dependent response of EECs to trophoblast derived EVs. Understanding the protein secretion dynamics in EECs in response to trophoblastic EVs may provide insights into the underlying mechanisms of implantation failure and offer potential improvements to assisted reproductive technologies. Furthermore, this in vitro model and MFGE8 read-out provides an effective platform to further study the mechanisms by which EVs influence their responding/target cells.

## Results

### MFGE8 protein secretion from EECs was affected by the estrogen and progesterone hormones

**Fig. 1:**
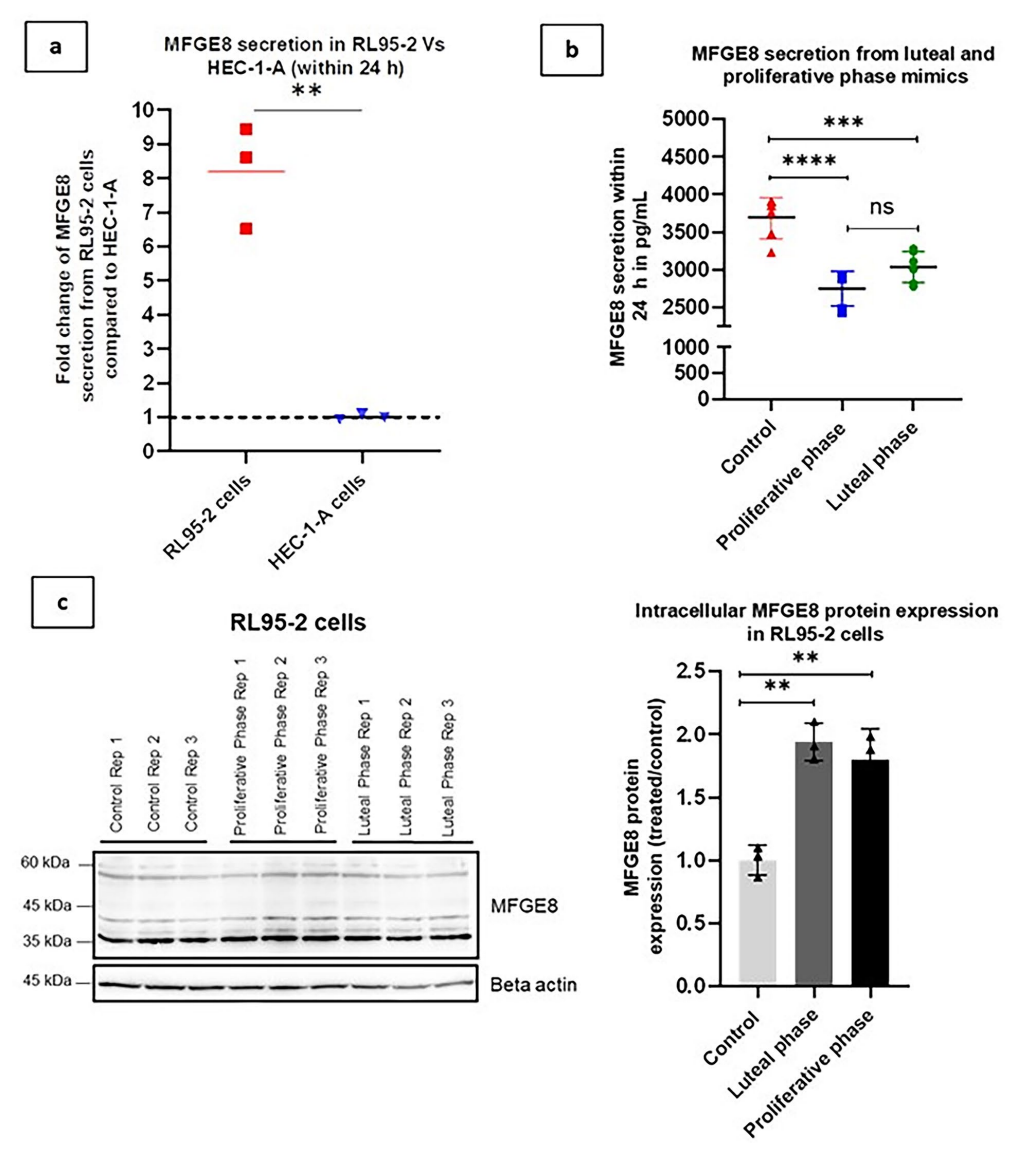
MFGE8 secretion from receptive and non-receptive EECs and the effects of estrogen and progesterone hormones **(a)** MFGE8 secretion from receptive EECs (RL95-2) vs. non-receptive EECs (HEC-1-A) **(b)** MFGE8 protein secretion in RL95-2 cells primed with combinations of estrogen and progesterone hormones that mimicked the luteal and proliferative phases of the menstrual cycle **(c)** Cropped western blot images and analysis showing intracellular MFGE8 protein expression in luteal and proliferative phase mimics of RL95-2 cells. β-actin was used as the loading control (stripped membranes were reprobed with β-actin). The results from three independent experiments were summarized as the mean ± SD (*n* = 3). Statistical significance was set at *P* < 0.05. **P < 0.01, ***P < 0.001, ****P < 0.0001. Original blots/gels are presented in Supplementary Fig. S3.

RL95-2 cells (receptive EEC analogue) secreted more MFGE8 protein than HEC-1-A cells (non-receptive EEC analogue) (*P* = 0.0055) (Fig. 1a). However, MFGE8 secretion was suppressed in RL95-2 cells under the influence of estrogen and progesterone hormone combinations that mimicked the luteal (*P* = 0.0008) and proliferative phases (*P* < 0.0001) of the menstrual cycle compared to the untreated controls (Fig. 1b). Conversely, RL95-2 cells displayed significantly higher intracellular MFGE8 protein expression in the follicular (*P* = 0.0029) and luteal phase mimics (*P* = 0.0012) than in untreated controls. No statistically significant alterations in intracellular MFGE8 protein expression were observed between luteal and proliferative phase simulations (Fig. 1c). The cell viability was not affected by hormone treatments (Supplementary Fig. S1).

### Trophoblast derived EVs increased MFGE8 secretion in both receptive and non- receptive endometrial epithelial cells.

**Fig. 2:**
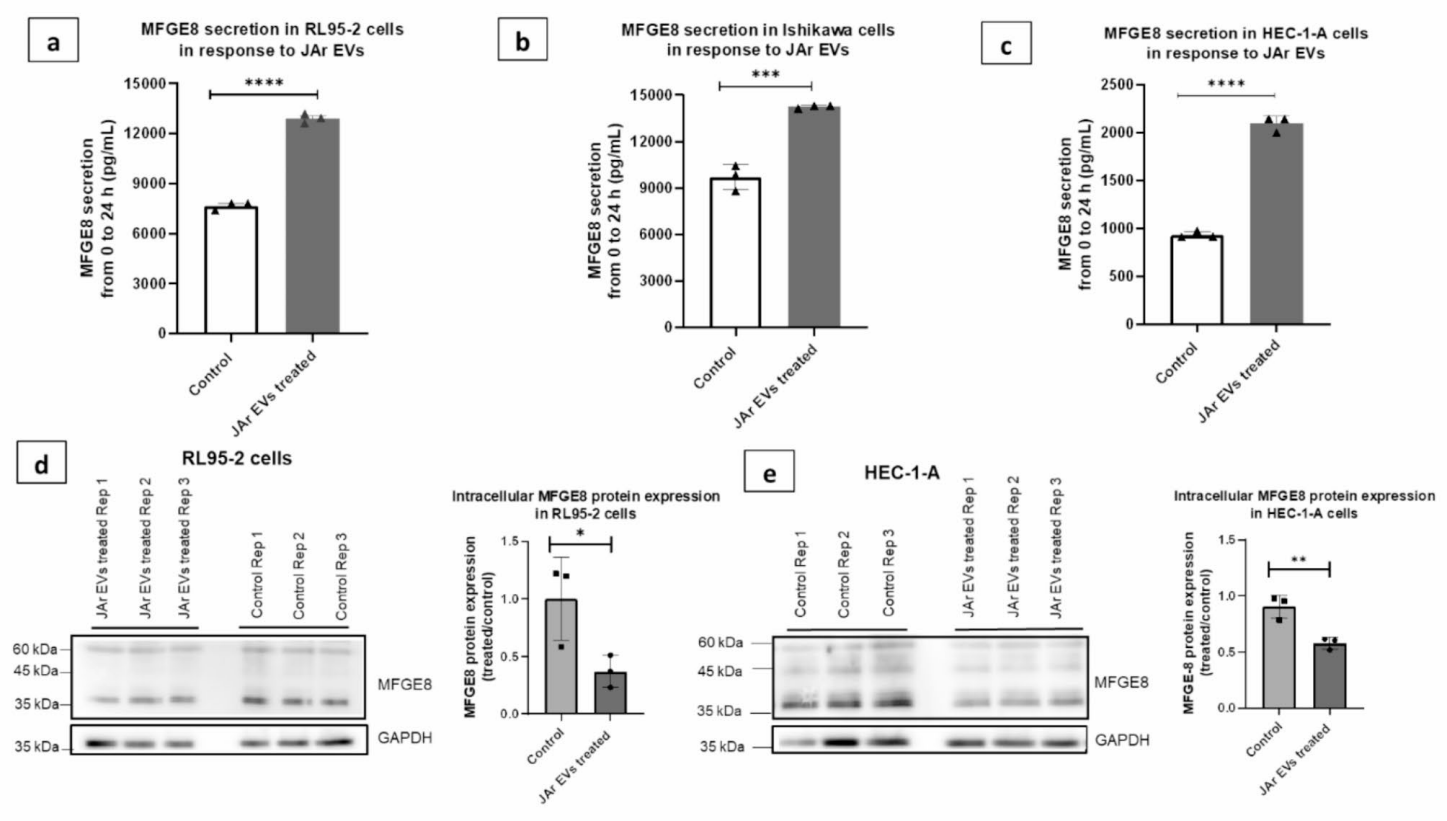
MFGE8 protein secretion from receptive and non-receptive EECs in response JAr- EVs treatment **(a)** MFGE8 protein secretion from RL95-2 cells in response to JAr-EVs **(b)** MFGE8 protein secretion from Ishikawa cells in response to JAr-EVs **(c)** MFGE8 protein secretion in HEC-1 A cells in response to JAr-EVs **(d)** Cropped western blot images and analysis showing intracellular protein expression in RL95-2 cells in response to JAr-EVs **(e)** Cropped western blot images and analysis showing intracellular protein expression in HEC-1 A cells in response to JAr-EVs. GAPDH was used as the loading control (stripped membranes were reprobed with GAPDH). The results from three independent experiments were summarized as the mean values ± SD (*n* = 3). Statistical significance was set at *P* < 0.05. *P < 0.05, **P < 0.01, ***P < 0.001, ****P < 0.0001. Original blots/gels are presented in Supplementary Fig. S3.

The amount of MFGE8 protein carried by JAr-EVs into the cell culture supernatants of RL95-2 cells and Ishikawa cells was determined to be negligible. Therefore, the presence of JAr-EV derived MFGE8 protein did not affect the measurements of MFGE8 secretion from EECs at the 24 h time point (Supplementary Fig. S2). Moreover, there were no statistically significant differences in cell viability between the JAr-EV treated and control groups (Supplementary Fig. S2). JAr-EVs significantly increased the secretion of MFGE8 protein from both receptive (RL95-2 and Ishikawa cells) and non-receptive EECs (HEC-1 A) within 24 h compared to PBS-treated controls (*P* = < 0.0001, *P* = 0.0007, *P* < 0.0001 respectively) (Fig. 2a and b, and 2c). Intracellular MFGE8 protein expression was significantly decreased in RL95-2 (*P* = 0.048) and HEC-1-A (*P* = 0.0082) cells in response to JAr-EVs within 24 h (Fig. 2d and e).

### MFGE8 protein secretion from EECs was time and dose dependent

**Fig. 3:**
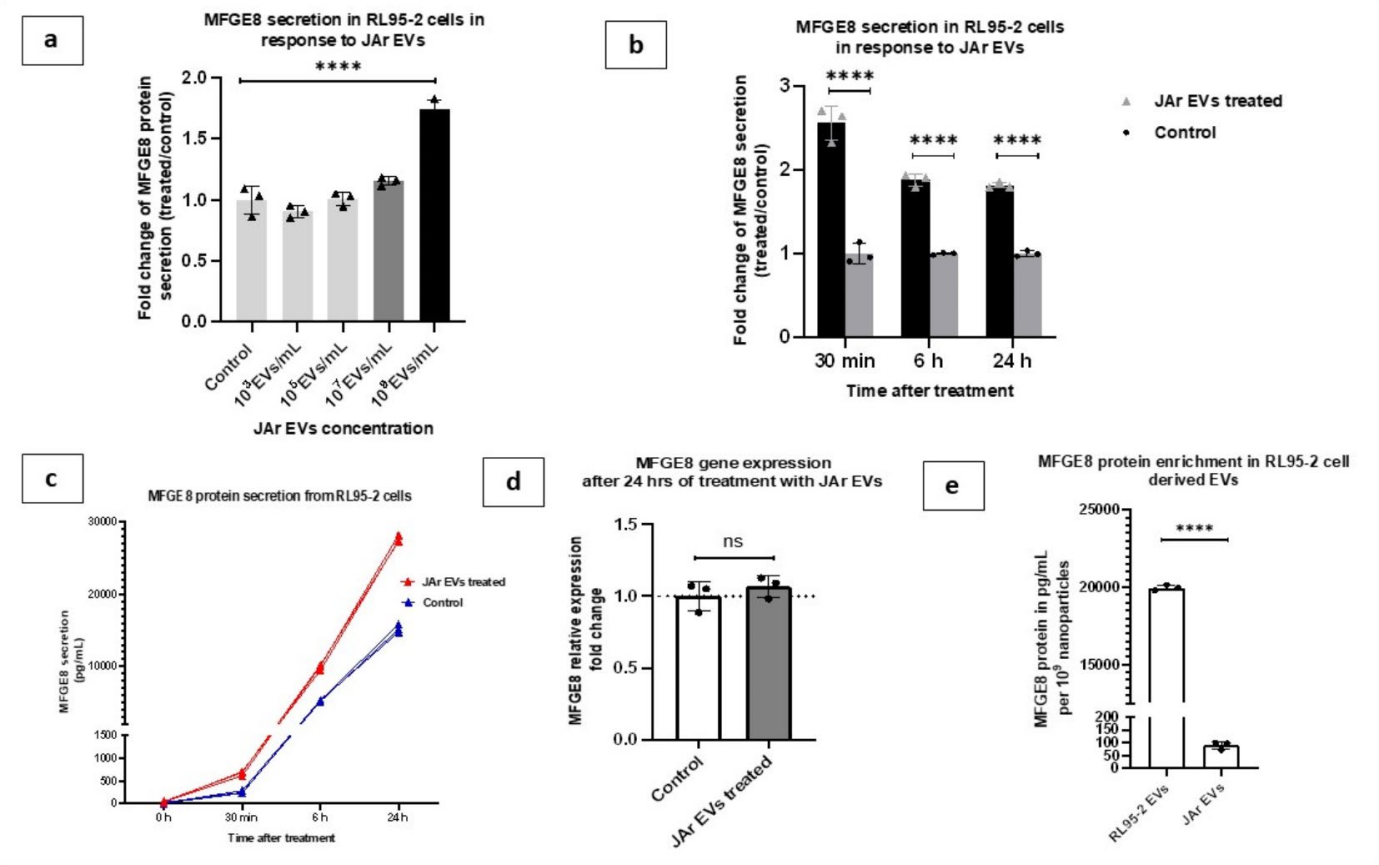
Dynamics of MFGE8 secretion from RL95-2 cells as a function of dose and time. **(a)** Fold change of MFGE8 secretion from RL95-2 cells as a function of JAr-EV concentration (Fold change of MFGE8 secretion was calculated compared to PBS treated control) **(b)** MFGE8 protein secretion from RL95-2 cells in response to JAr-EVs as a function against time **(c)** Absolute MFGE8 protein secretion values from RL95-2 cells in JAr-EVs treated and PBS-treated control groups in each time point **(d)** MFGE8 gene expression in RL95-2 cells in JAr-EVs treated and control groups after 24 h **(e)** MFGE8 protein enrichment in RL95-2 and JAr cell derived EVs. Results from three independent experiments were summarized with mean values ± standard deviation (SD) (*n* = 3). *P* < 0.05 considered statistically significant. ****P < 0.0001.

A minimum concentration of 10^9^ nanoparticles/mL of JAr-EVs was necessary to induce MFGE8 secretion from RL95-2 cells, equating to approximately 250 nanoparticles per cell (Fig. 3a). The secretion of MFGE8 in RL95-2 cells was significantly higher than that in the control group treated with PBS as early as 30 min after EV treatment (*P* = 0.00035) (Fig. 3b and c). However, MFGE8 gene expression remain unchanged after 24 h of JAr-EVs treatment (Fig. 3d). RL95-2 cell-derived EVs were highly enriched with MFGE8 protein compared to JAr cell derived EVs (*P* < 0.0001) (Fig. 3e).

## Discussion

There is a growing interest in understanding how EVs serve as mediators in embryo-maternal communication during human pre-implantation period. In our previous studies, we demonstrated that EV-mediated embryo-maternal communication during pre-implantation could potentially increase endometrial receptivity and prime the endometrium for the incoming embryo^17,19,21,22^. However, further understanding of the mechanisms governing EV-mediated embryo-maternal communication during this process would be beneficial for the treatment of infertility or the development of new contraceptive methods. In our ongoing efforts to elucidate this mechanism, we recently showed that EVs released by the embryo can induce multiple secretory proteomic changes in EECs, including an increase in MFGE8 protein secretion^22^. In the current study, we examined how MFGE8 secretion by EECs changes during embryo implantation to gain clearer insight into the potential mechanisms through which EVs induce alterations in secretory proteins. We observed higher MFGE8 secretion from the receptive EECs compared to the non-receptive EECs which is consistent with the previous findings that reported a relationship between MFGE8 protein expression and endometrial receptivity. According to previous reports, MFGE8 protein and gene expression in the endometrial epithelial compartment are elevated during the secretory phase of the menstrual cycle, with the greatest amount of expression occurring in the window of implantation^38^. When different cells in the endometrium during the window of implantation are considered, MFGE8 is mainly expressed in the epithelium of the endometrium, with no expression in the stroma, suggesting a potential role for MFGE8 protein, especially in modulating EECs for the incoming embryo^37,38^. Moreover, immunohistochemical analysis of human endometrial biopsies taken during the window of implantation revealed a marked elevation of MFGE8 protein in the apical cellular zones of the EECs, further supporting the luminal secretion of the protein. Therefore, it can be proposed that increased MFGE8 protein secretion might be required for the reprogramming of EECs towards embryo receptivity and attachment.

Endometrial receptivity is well known to be established through sequential exposure to high concentrations of two main steroid hormones, estrogen and progesterone which modify the endometrium for impending implantation^40,41^. Interestingly, when receptive EECs were exposed to a hormone combinations that mimicked the luteal and proliferative phases of the menstrual cycle, there was a notable decrease in the release of MFGE8 from cells compared to untreated controls. This lead to an accumulation of MFGE8 within the cells themselves^42^. Decreased MFGE8 secretion from hormone-treated RL95-2 cells could be a strategy used by the cells to increase intracellular MFGE8 protein expression, allowing for its release in response to signals from the embryo. MFGE8 is a ∼43 kDa secreted glycoprotein^43^ consisting of an N-terminal signal peptide that directs MFGE8 into the extracellular environment. However MFGE8 protein was reported to have short and long isoforms as well^44^. We identified multiple bands as specific to MFGE8 protein according to previous reports (due to high glycosylation at the post translational modification, the product sizes of each of these MFGE8 isoforms may vary within the ∼35 to 65 kD)^45,46^. Since all these isoforms retain their conserved binding domains intact, they exhibit similar function but with varied efficacy. Nevertheless, there was no significant change in MFGE8 protein expression or secretion between luteal and proliferative phase mimics in the current study. Therefore, MFGE8 protein secretion seemed to be affected by presence or absence of estrogen and progesterone hormones, but not by their alterations as it may happen during menstrual/reproductive cycle in the endometrium. However, research findings regarding the impact of estrogen and progesterone on MFGE8 protein expression in EECs have shown disparities. While some studies have reported that the combination of estrogen and progesterone does not influence MFGE8 production or secretion^35^, others have claimed that these hormones can activate the secretion of MFGE8 in EECs. The discordant results of these studies can be attributed to the use of different cell lines or tissues, different concentrations of estrogen and progesterone, and different experiment set ups. Another plausible explanation is that receptive EECs may already express high levels of the MFGE8 protein, hence, hormones have a minimal effect on increasing their expression in vitro and more studies are required to determine the dynamics of MFGE8 secretion under the influence of these hormones in both receptive and non-receptive EECs.

Interestingly, our data showed that embryonic signals in the form of EVs can increase the release of MFGE8 from both receptive and non-receptive EECs, which is in line with previous findings showing that embryonic signals such as TNFα or hCG can induce MFGE8 secretion in the receptive endometrium^47,48^. Conversely, the intracellular MFGE8 protein amount was decreased, suggesting the deployment of the protein from the cell. MFGE8 has previously shown to be required by EECs for embryo attachment^49^, and release of the MFGE8 protein to the peri-conception environment in response to embryonic signals can facilitate this process. The fact that embryonic EVs induced MFGE8 secretion from non-receptive EECs indicates that the EV mediated signalling can be very potent in reprogramming the EECs towards embryo reception. However, the overall role of MFGE8 secretion in embryo implantation can only be determined by the sum of the effects from EVs, hormones, and other soluble factors which require further studies.

The effect of embryonic EVs on receptive EECs was time and dose dependent. A minimum of 250 nanoparticle per cell was required to drive the effect of JAr-EVs on secretory protein changes in RL95-2 cells, which is a relatively a low EVs: cell ratio^50^. Few studies have documented that the biological effects of EVs can be dose and time dependent^51,52^. For instance, a study by Tabak et al., in 2018, showed that expression of canonical Wingless-related integration site proteins (Wnt) in normal trabecular meshwork cells can be impacted by the concentration of non-pigmented ciliary epithelium derived EVs^50^. However, based on our observation, it could be argued that the EVs to cell ratio is an important parameter in determining their mode of action. To date, many studies have focused less on the EV: cell ratio used in functional studies involving EVs. When considering the quantities of EVs used in various in vitro functional studies, researchers have employed anywhere from approximately 0.5–50 µg of EV proteins or a range of 10^9^ − 10^12^ nanoparticles per 1 × 10^6^ cells^53–55^. This wide variation in EV quantities translates to a significant disparity in the number of EV sized particles required to elicit biological effects in recipient cells, with some studies suggesting the need for as few as tens of EVs per cell^21,56^, whereas others indicate the necessity of hundreds of thousands of EVs per cell^57–59^. This is particularly interesting as there are discrepancies in effective EV dose used in various in vivo EV studies where doses ranged from 0.001 to 100 mg EV protein per kg body weight^59,60^. Apparently in many of these studies, EV doses have been decided without the taking in to account the pre-clinical studies on EV bio distribution or pharmacokinetics. Our results emphasized the value of exact EV number effect per cell. Another aspect that often goes unnoticed or receives inadequate attention is the EV quantification method. In most instances, EV quantification is performed in micrograms of proteins instead of particle numbers. This method frequently leads to an overestimation of vesicle numbers due to the presence of co-isolated protein contaminants in the EV preparations^59^. Some other studies report EV concentration in µg/ mL^20^, which makes the calculation of EV: cell ratio difficult^20^. With the development of more advanced EV purification methods, such as size-exclusion chromatography (which results in EVs of high purity compared to ultracentrifugation or filtration-based methods) and nanoparticle tracking analyser-based single EV quantification, we are now able to count the EV number as particles numbers more accurately. Therefore, we suggest that in the future, EVs number to cell ratio should be established as an important parameter in reporting EV effects in in vitro models.

We observed significant changes in MFGE8 secretion from RL95-2 cells treated with JAr-EVs at all three time points tested. It is interesting to note that the alteration of MFGE8 secretion takes place as early as 30 min of post EV treatment, suggesting that signal transfer between the embryo and the endometrium via EVs can be very quick. Cellular signalling based on EVs begins by targeting and binding of EVs to a cell^25^. This process has several steps; targeting the cell, binding to it, followed by the release of cargo content through cellular uptake^26,27^ or fusion^28,29^ which ultimately leads to signal transmission. However, EV internalization or all of the steps mentioned above are not always required for EV mediated responses. One potential explanation for the rapid release of MFGE8 protein from EECs is that EV bind to specific receptors in EECs that trigger intracellular signalling pathways^61^. Alternatively, it could be due to the release of EV cargo content by fusion of trophoblast-EVs with the EECs plasma membrane. EV binding or fusion to recipient cells does not require EV internalization for cargo release, and it can be hypothesized that the timing and mechanisms of membrane bound or fused EV processing may differ from that undergoes endocytosis for cargo release^28,29,62^. Interestingly, we observed that MFGE8 gene expression in EECs remained unchanged after 24 h of EV treatment. Additionally, in a recent study, we detected the transcriptomic changes in RL95-2 cells in response to JAr EVs at 30 min, 4 h, and 48 h time points. The expression of the MFGE8 gene remained unchanged at all time points^21^. The conventional protein secretion that happens under the control of gene transcription is a slow process. There the gene transcription is usually followed by protein translation and transportation from the endoplasmic reticulum to the plasma membrane^63^. Therefore, it is unlikely that the increased MFGE8 secretion was caused by the increased synthesis of the MFGE8 protein by transcriptional activation^64,65^. The most plausible explanation for these observations is the hypothesis that EVs engage in membrane signalling, fusion, or internalization processes. These interactions may result in the release of MFGE8 protein, which is stored inside the cells. However, increased MFGE8 secretion at later time points 6 h and 24 h could have a major contribution from EV internalization (as EV internalization increases over time)^51,66–68^. Increased EV internalization can lead to compensatory release of EVs by exocytosis^69^. Nevertheless, experimental evidence of EV membrane binding or fusion and its functional effects is still limited and inconclusive owing to the lack of a suitable read-out system^70^. EV binding or fusion with the recipient cell membrane is an extremely rapid process occurring within milliseconds and increasing over time^28^. Fluorogenic dequenching assays, fluorescence time-lapse imaging, electron microscopy, and spectrofluorimetry-based methods have been used to detect EV docking, binding, and fusion with recipient cell membranes, typically taking less than 10 min for a detectable signal^28,29,62^. Nevertheless, most of the above methods lack the ability to detect the functional effects of EV fusion in recipient cells. Therefore, if a relationship between EV membrane binding, fusion, or internalization and MFGE8 release can be established, this in vitro model system of embryo implantation and MFGE8 read-out hold promise as a unique system to further study the natural mode of EV action. The above observations on the effect of EV dose and time on MFGE8 protein release are important in elucidating the exact molecular mechanism of EV function in the embryo maternal interface. Nevertheless, the EVs timing and dosing concentrations we used were not sufficient for analysing the full time and dose–response curves.

We found that MFGE8 protein was highly enriched in RL95-2 cell derived EVs compared to JAr-EVs. Our previous findings indicated that RL95-2 cell-derived EVs were notably rich in MFGE8 protein compared to their corresponding cell culture supernatants, further emphasizing the substantial enrichment of MFGE8 in RL95-2 EVs^23^. Two distinct hypotheses have been proposed to explain the secretion of MFGE8 from mammalian cells, which may be caused by exosome secretion from cells or by membrane shedding. MFGE8 is widely known to be a peripheral membrane-bound glycoprotein, which is also associated with nanovesicles of 100–200 nm in size and is secreted by cells into the extracellular space^71^. Hence, MFGE8 secretion in this context is likely a response of EV binding or fusion with recipient cell membrane that activates signalling pathways to release MFGE8 protein stored inside cells in the form of vesicles. Nevertheless, future studies are required to elucidate the exact mode of embryonic EV action on EEC MFGE8 release.

While there are increasing evidence that EVs are involved in embryo maternal interaction in pre-implantation, the exact mechanism of EV function remain elusive. In the current study we were able to establish a potential indicator of EV mediated embryo maternal dialogue. Previously, studies identified transcriptomic and proteomic changes of EECs to trophoblastic EVs suggesting the potential for using them as mRNA and protein markers to further decipher EV mechanism of action^19,20^. However, the correlation between mRNA and protein expression is variable^72,73^ and biological processes are mainly driven by proteins. Measuring proteins in the cell culture supernatants, which could reflect EV function, can eliminate the need for mRNA or protein extraction steps, making them more convenient markers of EV function in this context. Effect of different EV uptake or binding inhibitors on MFGE8 secretion from EECs could be used to decipher the exact mechanism of action of EVs on recipient cells. The in-vitro model of embryo implantation consisting of JAr and RL9-5 or Ishikawa cells has been used since 1970s to represent preimplantation microenvironment, however, confirming these findings in an in vitro model consisting with primary cells will further strength these findings for enhancing their potential for clinical translation. Overall, findings of this study hold promise for the deciphering EV mechanism of action on recipient cells and in development of novel diagnostic and therapeutic tools to enhance assisted reproduction technologies in the future.

## Conclusion

In conclusion, this study revealed the involvement of estrogen and progesterone hormones in increasing the MFGE8 protein levels in EECs. Interestingly, trophoblast derived EVs were able to stimulate EECs to release the MFGE8 protein, suggesting that MFGE8 protein secretion from both receptive and non-receptive EECs is a response to embryonic cues. The effects of trophoblastic EVs on MFGE8 protein secretion by EECs were dose and time dependent. Specially, the secretion of MFGE8 increased within a short timeframe of 30 min after addition of EVs, indicating the occurrence of rapid processes such as binding, fusion, or internalization of EVs within recipient cells. These interactions likely trigger the release of MFGE8, stored within the cells in vesicular form. Therefore, this in vitro model and MFGE8 readout hold promise as a unique platform for studying the mechanism of EV function at the recipient cell level. A comprehensive understanding of protein secretion dynamics in EECs in response to trophoblastic EVs may provide insights into the underlying mechanisms of EV mediated embryo maternal communication preceding implantation.

## Materials and methods

### Cell culture

The human endometrial cell lines, RL95-2, HEC-1 A and human trophoblast cell line, JAr cells were purchased from the American Type Culture Collection. Ishikawa cell line was purchased from European Collection of Authenticated Cell Cultures. RL95-2 and JAr cells were grown as described previously^22^. In summary, RL95-2 (ATCC CRL-1671, Teddington, UK), Ishikawa (ECACC 99040201) and HEC-1 A cells (ATCC^®^ HTB-112™, Teddington, UK) were grown in Dulbecco’s Modified Eagles medium F12 (DMEM 12–604 F, Lonza, Verviers, Belgium) consisting of 10% Fetal Bovine Serum (FBS) (Gibco™, 10500064), 1% penicillin streptomycin (P/S) (Gibco™ 15140122, Bleiswijk, Netherlands) and 5 µg/ml Insulin (human recombinant insulin, Gibco, Invitrogen, Denmark) in 5% CO_2_ at 37^o^C. JAr cells (HTB-144™, Teddington, UK) were grown in RPMI 1640 medium supplemented with 10% FBS, 1% L- glutamine and 1% P/S in 5% CO_2_ at 37^o^C. The medium was changed every second day until the cells reached 80% confluency. For hormone study, RL95-2 cells were grown in DMEM F12 medium supplemented with 10% FBS, 5 µg/ml Insulin, and 5% P/S. At 70% confluency, RL95-2 cell culture media were replaced with phenol red free DMEM F12 medium (Gibco™ 21041033) supplemented with 5% P/S (serum free) and incubated for 24 h in 5% CO_2_ at 37^o^C. After 24 h, appropriate hormone treatments were added to the cells in phenol red-free DMEM culture media supplemented with 5% charcoal stripped FBS.

### Preparation of EV depleted media

EV depleted medium was prepared using EV depleted fetal bovine serum (FBS) as previously described^19,22^. In summary, FBS was filtered using Amicon^®^ Ultra-15 centrifugal filters with a 100 kDa cut-off (MERCK KGAA, Darmstadt, Germany) at 3000× g for 55 min. The filtered FBS used to prepare EV depleted media was 90% depleted of nanoparticles and used at 10% concentration to supplement all complete culture media specific to each cell type mentioned above.

### EV isolation and characterization

At 80% confluency, JAr and RL95-2 cells were washed with phosphate buffered saline (PBS) and culture conditioned media were replaced with EV depleted culture media. After 24 h, the cell culture supernatants were collected and centrifuged at 400× g for 10 min to remove contaminating cells. The resulting supernatant was centrifuged again at 4000× g for 10 min, followed by centrifugation at 10,000× g for 10 min to remove remaining cellular debris and apoptotic bodies. The collected media was concentrated to a final volume of 500 µl using Amicon^®^ Ultra-15 centrifugal filters with a 10 kDa cut-off. Next, EVs were purified using size exclusion chromatography (SEC). A gel filtration medium consisting of 4–6% agarose matrix was used in 15 cm length columns to separate EV fractions from contaminating proteins. Fractions 7–10 were collected (each fraction was 500 µl in volume) and concentrated again to a total volume of 500 µl using a Amicon^®^ Ultra centrifugal filter device with a 10 kDa cut-off. Characterisation of the isolated EVs was carried out using methods described in detail elsewhere^17,19,75^. In summary, the nanoparticle size and concentration in EV fractions were measured using Nano Particle Tracking Analyser (Particle Metrix GmbH, Inning am Ammersee, Germany). Transmission electron microscopy was used for the physical characterization of EVs. Enrichment of EV surface protein markers, CD 9, CD 63, and CD 81 was confirmed by western blotting or label free proteomic analysis. The methods used for cell culture derived EV characterization were fully described in our previous publications^17,19,23,75^. EVs derived from JAr cells are referred to as JAr-EVs. EVs from RL95-2 cells are referred to as RL95-2 EVs.

### MFGE8 enzyme linked immunosorbent assay

MFGE8 protein concentration was measured in cell culture supernatants using a commercially available ELISA kit (Human MFGE8 Quantikine ELISA Kit, R&D systems) according to the manufacturer’s instructions. In summary, cell culture supernatants (1 ml of media) were centrifuged at 400× g for 10 min to remove any contaminating cells, followed by 4000× g for 10 min and 10,000× g for 10 min to further remove other contaminating cellular debris. Samples were snap frozen in liquid nitrogen before storing in -80^o^C. Protocol for MFGE8 ELISA was described in our previous publication in detail^22^.

### Western blotting

After collecting cell culture supernatants from the cells grown in 12 well plates for MFGE8 ELISA, the cells were washed once with ice cold PBS. Then, total proteins were isolated by lysing the cells in 250 µl of radio immunoprecipitation buffer (RIPA Lysis and Extraction Buffer, Thermo Scientific™, Rockford, USA**)** with protease inhibitors. After centrifugation of the cell lysate at 15,000 *g* for 5 min at 4 °C, the supernatants were collected. Protein concentrations were determined by Bradford assay (Pierce 23246) and used to normalize all samples. The samples were then heated to 95 °C for 5 min in SDS-PAGE loading buffer, separated by SDS-PAGE, and transferred to polyvinylidene difluoride (PVDF) membrane (Thermo Scientific™, Rockford, USA). The membranes were blocked in 5% milk in phosphate buffered saline with 0.1% Tween 20 (PBST) and incubated overnight with primary antibodies for GAPDH (1:1000, Santa Cruz, sc-47724), Beta-actin (1:5000, Proteintech, 20536-1-AP) and MFGE8 (1:1000, SAB1408603, Sigma-Aldrich) in 5% milk-PBST solution and then with horseradish peroxidase conjugated goat anti-mouse (sc-516,102, 1:10000, Santa Cruz Biotechnology Inc. Dallas, TX, USA) or goat anti-rabbit (sc-2357, 1:10000, Santa Cruz Biotechnology Inc. Dallas, TX, USA) secondary antibody for 2 h at room temperature. Membranes were washed three times for 5 min in PBST after each incubation step. Protein bands were detected using ECL Select™ Western Blotting Detection Reagent following manufacturer’s protocols (GE Healthcare, Buckinghamshire, UK) with ImageQuant™ RT ECL Imager (GE Healthcare, Buckinghamshire, UK). Western blots were quantified using densitometry in image J software.

### RNA extraction and MFGE8 RT-PCR

Total RNA was enriched from cells using TRIzol reagent (Invitrogen, 15596018) and ethanol precipitation. The RNA content in samples was quantified using a nanodrop. Total RNA was reverse transcribed in 20 µl reactions using FIREScript^®^ RT cDNA synthesis Kit (Solis BioDyne, Tartu, Estonia) and as described previously^21^. Primers for MFGE8 gene was designed in NCBI primer blast and the primer efficiency was tested (R^2^ = 0.9956) (Primer pairs used in the study are listed in Supplementary Table S1). MFGE8 RT-PCR was performed using HOT FIREPol^®^ EvaGreen^®^ qPCR Supermix (Solis BioDyne, Estonia) with the following settings: 95 °C for 15 min; 40 cycles of 95 °C for 20 s; 60 °C for 20 s, and 72 °C for 20 s. Finally, melting curve was obtained by continuously collecting fluorescence data while slowly heating the samples from 65 °C to 95 °C at 0.05 °C per second rate. Cycle thresholds (Ct) were normalized to the Ct of β-2 microglobulin. The 2^−∆∆Ct^ method was used to calculate the relative quantification of the MFGE8 gene expression^76^.

### Cell viability assessment using Trypan blue exclusion method

Cells were washed with DPBS, dissociated and pelleted using Trypsin/EDTA solution (Sigma-Aldrich^®^, USA). The cell pellet was re-suspended in growth medium and then a portion of cell suspension was mixed with trypan blue. The viable cell count in original wells was determined using the haemocytometer cell counting method as described earlier^77^.

### Experimental design

#### MFGE8 secretion from receptive vs. non receptive EECs

MFGE8 secretion from receptive vs. non-receptive EECs was determined using RL95-2 and HEC-1 A cells as models of receptive and non-receptive EECs. RL95-2 and HEC-1 A cells were seeded in 12 well plates (0.1 × 10^6^ cells/ml) and grown until they reached 85% confluency. After reaching the desired confluency, the cells were washed with Dulbecco’s phosphate-buffered saline without Ca^+2^ and Mg^+2^ (DPBS, Verviers, Belgium). Then, the medium was replaced with EV-depleted medium, and cell culture supernatants were collected after 24 h and MFGE8 concentration was measured using MFGE8 ELISA. Viable cell count at the time of collection of the cell culture supernatant was performed using the trypan blue exclusion method and used to normalize MFGE8 secretion within 24 h. Experiments were performed in triplicates and data were presented as mean ± Standard Deviation (SD).

#### Stimulation of RL95-2 cells with estrogen and progesterone hormone combinations that mimicked main phases of the menstrual cycle.

Intracellular and secretory MFGE8 protein expression in RL95-2 cells stimulated with oestrogen and progesterone hormone combinations that mimicked the follicular and luteal phases of the menstrual cycle were investigated as below. RL95-2 cells were seeded in 12 well plates (0.1 × 10^6^ cells/ml) and grown until 70% confluency. Cells were then serum-starved for 24 h in phenol red free medium, followed by hormone treatment in phenol red free medium supplemented with charcoal stripped FBS for 24 h (as described in 2.1). To mimic the receptive phase of the menstrual cycle, estrogen and progesterone were added at final concentrations of 10^−8^ M 17β-estradiol (Acros Organics, Geel, Belgium) and 10^−6^ M progesterone (Acros Organics, Geel, Belgium). To mimic the proliferative phase of the menstrual cycle, 10^−7^ M 17β-estradiol and 10^−8^ M Progesterone were used, and the concentrations were adapted as described previously^6,20,35^. Next, cell culture supernatants were collected, and secretory MFGE8 concentration was measured using ELISA. Cell lysates were collected for western blotting, and changes in intracellular MFGE8 protein levels were measured as well. The cell viability after hormone treatment was measured using the trypan blue exclusion method. Hormone-treated groups were compared with each other and with untreated controls. Experiments were performed in triplicate, and data were presented as the mean ± SD.

#### Stimulation of EECs with trophoblast derived EVs and measuring MFGE8 gene and protein expression

MFGE8 secretion from EECs in response to trophoblast-derived EVs was determined in three endometrial epithelial cell lines. RL95-2 and Ishikawa cells were used as the analogs of the receptive EECs. HEC-1-A cells were used to mimic non-receptive EECs. RL95-2, Ishikawa, and HEC-1-A cells were seeded in 12 well plates (1 × 10^5^ cells/ml) and grown until they reached 85% confluency. After reaching the desired confluency, the cells were washed with DPBS. All cell types were supplemented with EVs derived from JAr cells at a concentration of 1 × 10^9^ nanoparticles/ml in EV-depleted medium, incubated for 24 h and cell culture supernatants were collected for MFGE8 ELISA. Cell culture supernatants were also collected 0 h after EV treatment to assess the level of MFGE8 protein originating from EV treatment. MFGE8 protein secretion from RL95-2, Ishikawa, and HEC-1-A cells was calculated by deducting the 0 h MFGE8 protein from the 24 h MFGE8 protein levels in the cell culture supernatants to further minimize the effect of MFGE8 derived from JAr-EVs. Cell viability in the treated and control groups was assessed after 24 h using the trypan blue exclusion method to verify that the increase in MFGE8 expression was not caused by changes in cell viability or proliferation.

The Minimum number of trophoblastic EVs required per cell to induce MFGE8 secretion from RL95-2 cells was determined by supplementing RL95-2 cells with EVs derived from JAr cells at five different concentrations (0, 1 × 10^3^, 1 × 10^5^, 1 × 10^7^, 1 × 10^9^ nanoparticles/ml) in EV-depleted medium, and cell culture supernatants were collected after 24 h to measure the secretory amount of MFGE8 using ELISA. PBS treated cells were used as controls. The number of cells after 24 h of EV treatment was determined and used to calculate the EVs to cell ratio for each treatment group.

The time required to induce MFGE8 secretion from RL95-2 cells was determined by incubating RL95-2 cells with JAr-EVs for 30 min, 6 h, and 24 h. Cell culture supernatants were collected for MFGE8 measurements. Controls were treated with PBS at each time point and used for comparison. After collecting the cell culture supernatants, they were stored at -80^o^C. Cell lysates were also collected, and intracellular MFGE8 protein expression was measured by western blotting. For mRNA expression analysis, RL95-2 cells were seeded in 12 well plates (1 × 10^5^ cells/ml) and grown until they reached 85% confluency. After reaching the desired confluency, cells were washed with DPBS. Then cells were supplemented with EVs derived from JAr cells at a concentration of 1 × 10^9^ nanoparticles/ml in EV-depleted medium. Total RNA was isolated, and RT-PCR was performed. PBS-treated cells were used as the controls. The cell counts in the treated and control groups were determined using the trypan blue exclusion method. All the experiments were performed in triplicate and data were presented as mean ± SD.

#### Determining the form of MFGE8 secretion from EECs

To investigate whether the MFGE8 released by RL95-2 cells is linked to the EVs they secrete, we measured the amount of MFGE8 protein in 10^9^ nanoparticles in RL95-2 cell-derived EV preparations using ELISA. Similar amounts of JAr-EVs were used as a control EV source. Data presented as mean ± SD. All experiments were performed in triplicates.

### Statistical data analysis

Data analysis was performed using GraphPad Prism 8.0 (GraphPad Software, La Jolla, CA, USA). Statistical differences were assessed by t-test for comparing two groups, one-way analysis of variance (ANOVA) with Tukey’s post-hoc test or Dunnet’s test was used for comparing multiple groups. Normality of the data were tested with Shapiro-Wilk normality test before group comparisons. Data were presented as mean plus/minus SD (mean ± SD). Statistical significance was set at *P* value < 0.05.

## Electronic supplementary material

Below is the link to the electronic supplementary material.

Supplementary Material 1

## Data Availability

All data generated or analysed during this study are included in this published article and its supplementary information files (uncropped western blot images are shown in Supplementary Fig. S3).
