# Supplementary material for: Rapid increase of MFGE8 secretion from endometrial epithelial cells is an indicator of extracellular vesicle mediated embryo maternal dialogue
Source: Sci Rep. 2024 Oct 29;14:25911. doi: 10.1038/s41598-024-75893-1 (PMC11522515; doi:10.1038/s41598-024-75893-1)
Supplement: Supplementary file 1 — Supplementary Material 1 [file 41598_2024_75893_MOESM1_ESM.docx]

Rapid increase of MFGE8 secretion from endometrial epithelial cells is an indicator of extracellular vesicle mediated embryo maternal dialogue

Subhashini Muhandiram, Suranga Kodithuwakku, Kasun Godakumara, Alireza Fazeli

**Supplementary Table S1.** Primer pairs

| **Transcript name** | **Forward primer** | **Reverse primer** |
| --- | --- | --- |
| MFGE8 | GTTTGAGACCCCTGTGGAGG | GATTGGCGCATCCGTTCAG |
| B2M | CGGGCATTCCTGAAGCTGA | TGGAGTACGCTGGATAGCCT |

**Supplementary Figure S1:** Cell viability after hormone treatment

**Supplementary Figure S1.** Bar chart showing the viable cell count in RL95-2 cells after treating with oestrogen and progesterone hormone combinations that mimic the luteal and proliferative phase of the menstrual cycle

**Supplementary Figure S2.** JAr EVs doesn’t affect MFGE8 measurements or cell viability of endometrial epithelial cells


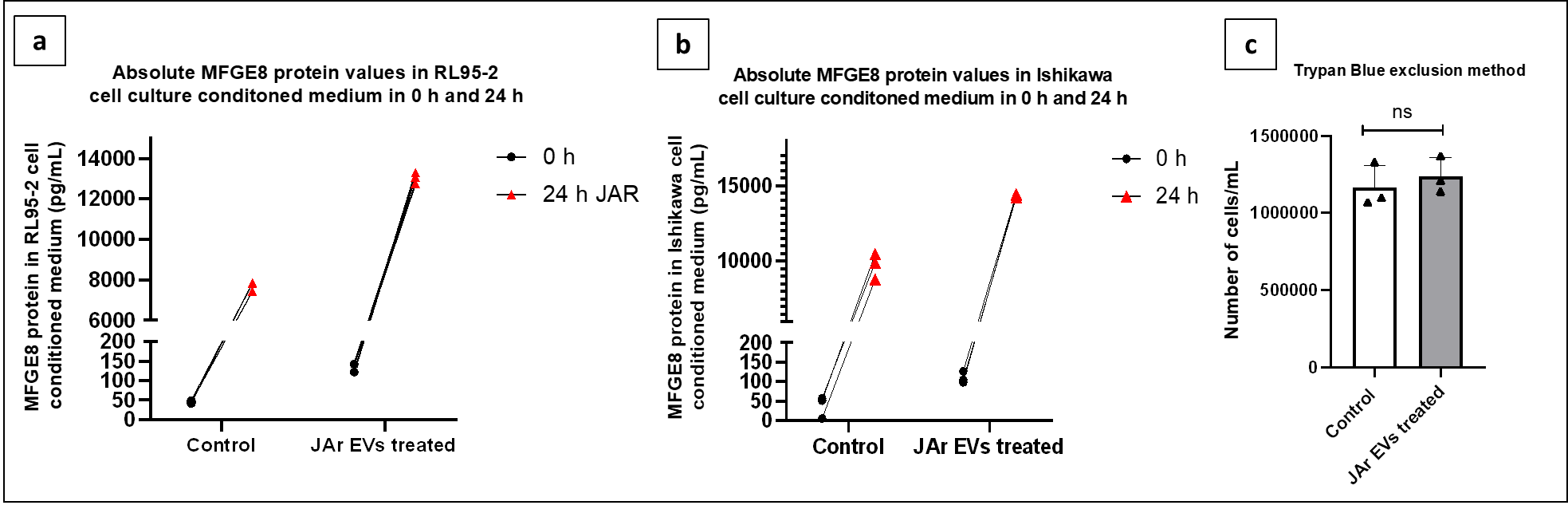


**Supplementary Figure S2a.** Absolute MFGE8 protein concentration in RL95-2 cell culture conditioned medium in 0 h and 24 h after treatment with JAr EVs **S2b.** Absolute MFGE8 protein concentration in Ishikawa cell culture conditioned medium in 0 h and 24 h after treatment with JAr EVs **S2c.** Bar chart showing the viable cell count in RL95-2 cells after treating with JAr EVs for 24 h.

**Supplementary Figure S3**. Uncropped blots related to Figure 1 and 2

**Uncropped blots related to Figure 1c**

**
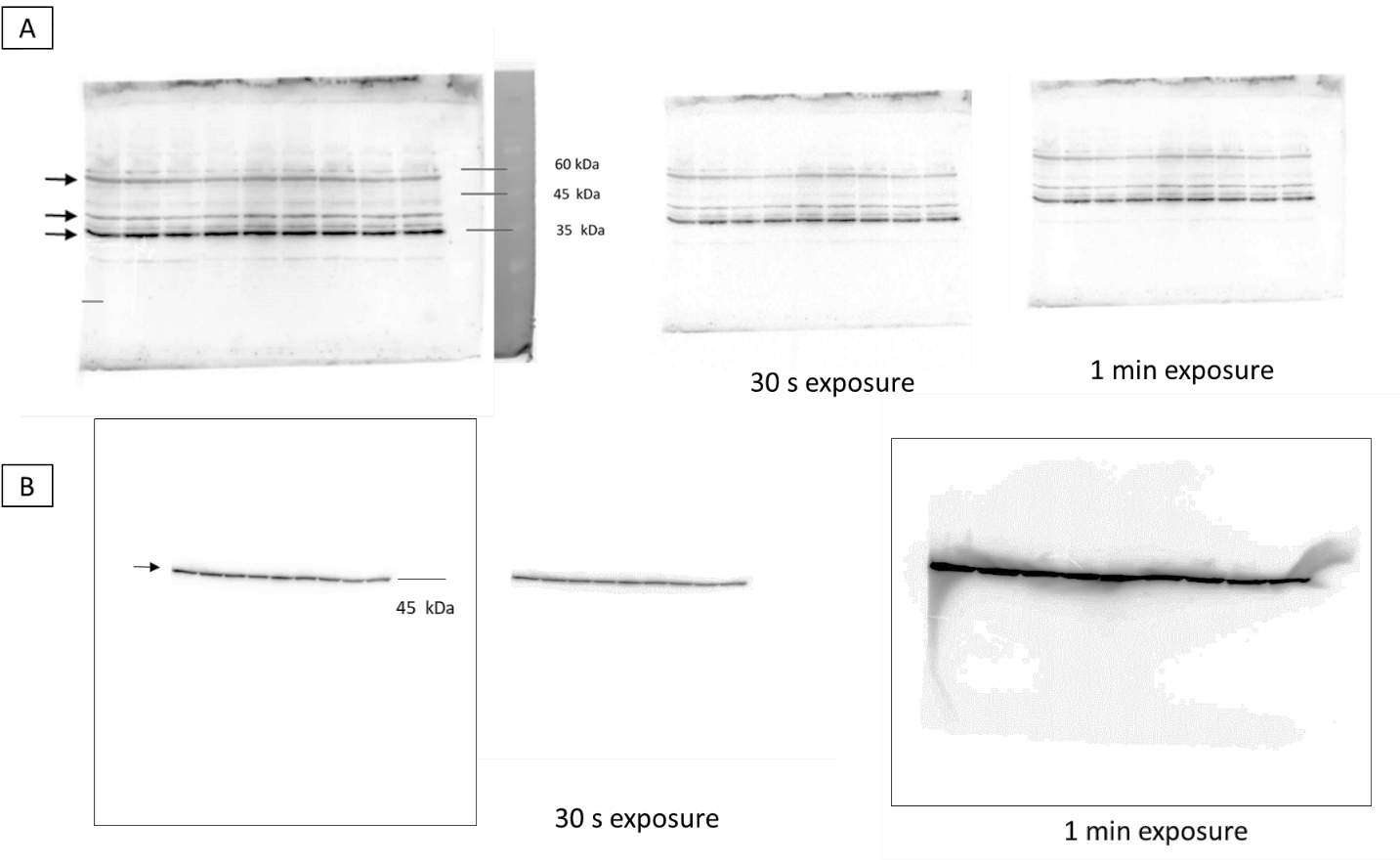
**

Uncropped western blot images showing A) Intracellular MFGE8 expression in luteal and proliferative mimics of RL95-2 cells. B) Loading control were run by stripping and reprobing the same membrane with β-actin antibody. Membranes at different exposure times were also shown. The bands considered for quantifying the intensities were marked by black arrows.

**Uncropped images related to Figure 2d**

**
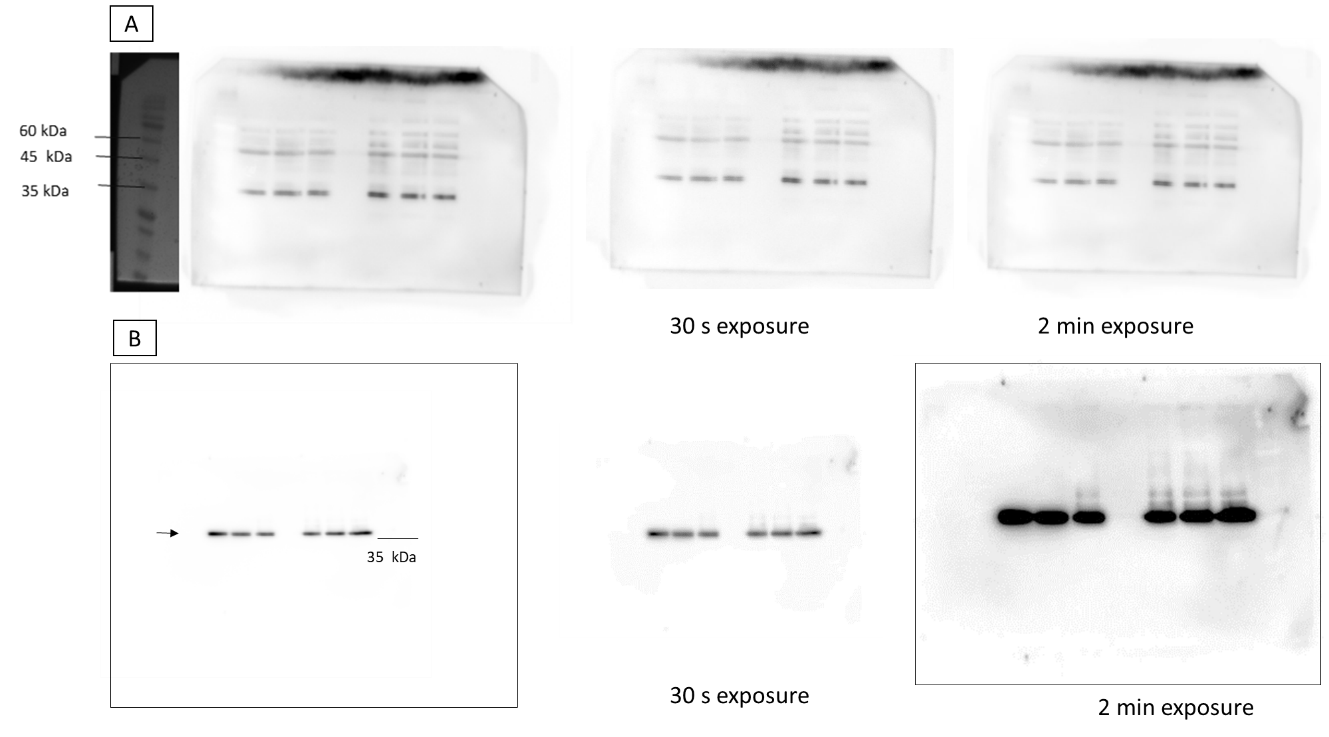
**

Uncropped western blot images showing A) intracellular MFGE8 expression in RL95-2 cells treated with JAr EVs Vs control. B) Loading control were run by stripping and reprobing the same membrane with GAPDH antibody. Membranes at different exposure times were also shown. The bands considered for quantifying the intensities were marked by black arrows.

**Uncropped images related to Figure 2e**


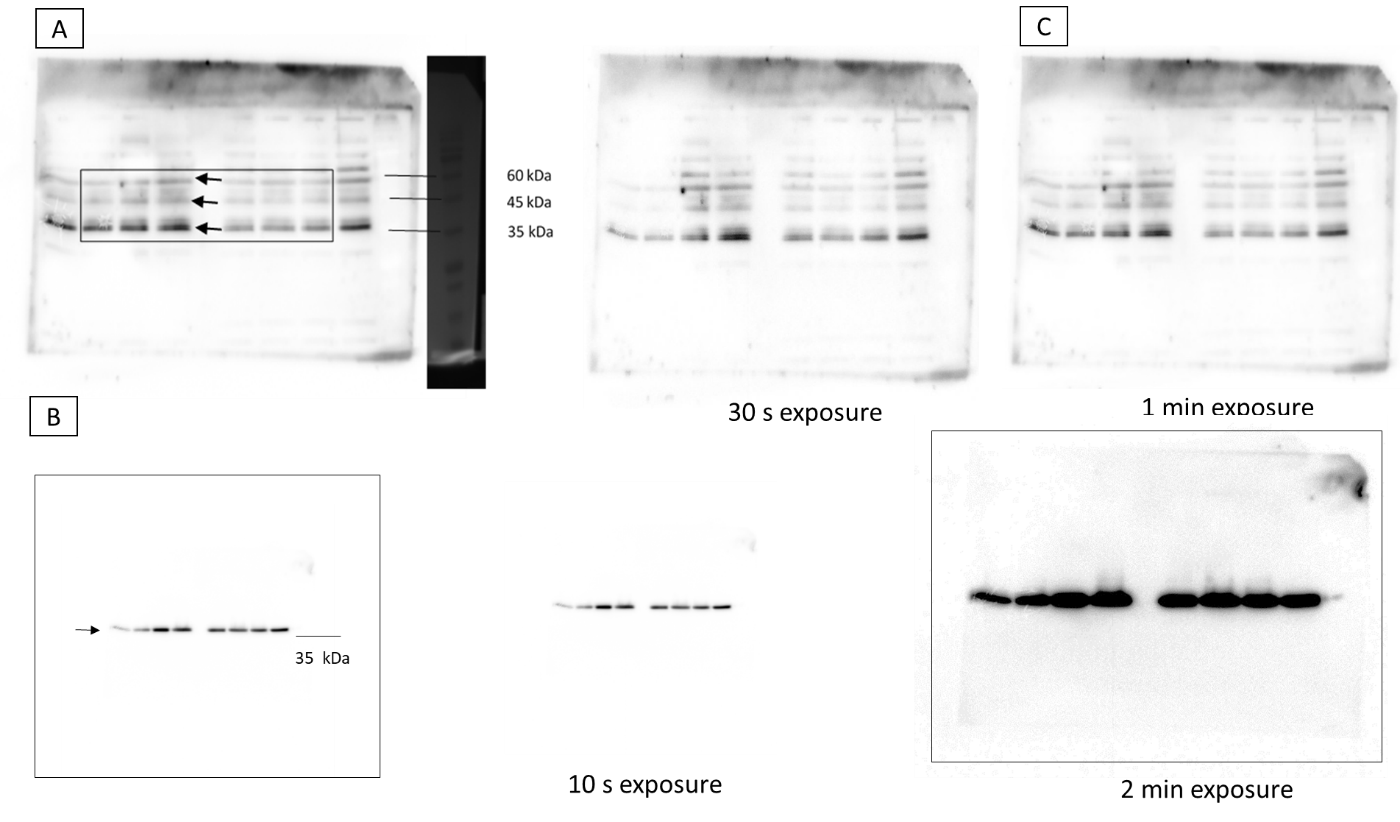


Uncropped western blot images showing A) intracellular MFGE8 expression in HEC-1A cells treated with JAr EVs Vs control. B) Loading control were run by stripping and reprobing the same membrane with GAPDH antibody. Membranes at different exposure times were also shown. Experiments had four replicates. Three replicates inside the black box were visualized in the main manuscript figure. The bands considered for quantifying the intensities were marked by black arrows. The images at different exposure time was shown.
